# Transformation of a Benign Adrenocortical Adenoma to a Metastatic Adrenocortical Carcinoma Is Rare But It Happens

**DOI:** 10.1210/jcemcr/luae131

**Published:** 2024-07-30

**Authors:** Anna Angelousi, Anne Jouinot, Charis Bourgioti, Panagiotis Tokmakidis, Jérôme Bertherat, Gregory Kaltsas

**Affiliations:** First Department of Internal Medicine, Unit of Endocrinology, Laikon Hospital, Center of Excellence of Endocrine Tumours, National and Kapodistrian University of Athens, 11527 Athens, Greece; Université de Paris, Institut Cochin, Department of Endocrinology, Referral Center for Rare Adrenal Diseases, INSERM U-1016, CNRS UMR- 8104, 75014 Paris, France; Department of Radiology, School of Medicine, National and Kapodistrian University of Athens, Aretaieion Hospital, 11528 Athens, Greece; Neuroendocrine Tumor Unit, ENETS Centre of Excellence, 1st Department of Propaedeutic and Internal Medicine, Laiko Hospital, National and Kapodistrian University of Athens, 11527, Athens, Greece; Université de Paris, Institut Cochin, Department of Endocrinology, Referral Center for Rare Adrenal Diseases, INSERM U-1016, CNRS UMR- 8104, 75014 Paris, France; Neuroendocrine Tumor Unit, ENETS Centre of Excellence, 1st Department of Propaedeutic and Internal Medicine, Laiko Hospital, National and Kapodistrian University of Athens, 11527, Athens, Greece

**Keywords:** adrenal incidentalomas, adrenocortical carcinoma, adrenal adenoma, adrenal incidentaloma, malignant transformation, malignant evolution

## Abstract

The transformation of an adrenocortical adenoma (ACA) to an adrenocortical carcinoma (ACC) is extremely rare. Current guidelines suggest against further imaging studies and follow-up in patients with nonfunctional adrenal incidentalomas (NFAIs) with benign imaging characteristics. Herein, we present a 64-year-old male patient diagnosed initially with a NFAI of 3 cm in size with imaging characteristics consistent with an ACA. However, 13 years after initial diagnosis, this apparent ACA developed into a high-grade cortisol and androgen-secreting ACC with synchronous metastases. The literature review revealed a further 9 case reports of adrenal incidentalomas initially characterized as ACA that subsequently developed into ACC within a period ranging from 1 to 10 years. The pathogenesis of transformation of an initially denoted ACA to ACC is not fully delineated, although the existing literature focuses on the preexisting or changing genetic background of these lesions, highlighting the need to develop robust prognostic markers to identify patients at risk and individualize the follow-up of these unique cases.

## Introduction

The incidence of adrenal tumors discovered often on cross-sectional abdominal imaging ranges between 5% and 7% of patients, increasing with age (1). Over the last 2 decades, the incidence of adrenal tumors has increased 10-fold, in parallel to the increase of abdominal computerized tomography (CT) and magnetic resonance imaging (MRI) studies performed (2). Malignant transformation of an adrenal incidentaloma (AI) considered to be an adrenocortical adenoma (ACA) to an adrenocortical carcinoma (ACC) is extremely rare, with an incidence of less than 1% (2). However, this figure varies according to the size of ACAs, ranging from 2% for tumors of 4 to 6 cm to 25% for tumors >6 cm (3).

Recent guidelines recommend that all AIs should be evaluated initially with a noncontrast CT to estimate their lipid content as well as their appearance whether it is homogenous or not (4). If the noncontrast CT is consistent with a benign adrenal mass with a baseline density of <10 Hounsfield (HU), no further imaging is suggested. Although such an approach is cost-effective and reduces the burden of regular investigations along with the danger of radiation exposure, rare cases of ACCs that were initially diagnosed as ACAs have been described (5-13).

Herein, we report the case of a 64-year-old male patient diagnosed with a nonfunctional ACA that transformed (the process that a normal cell undergoes as it becomes malignant) into a metastatic ACC 13 years after initial diagnosis. An extensive review of the literature has also been performed including cases with an initial diagnosis of AI with benign characteristics that subsequently transformed to ACC.

## Case Presentation

**Figure 1.**
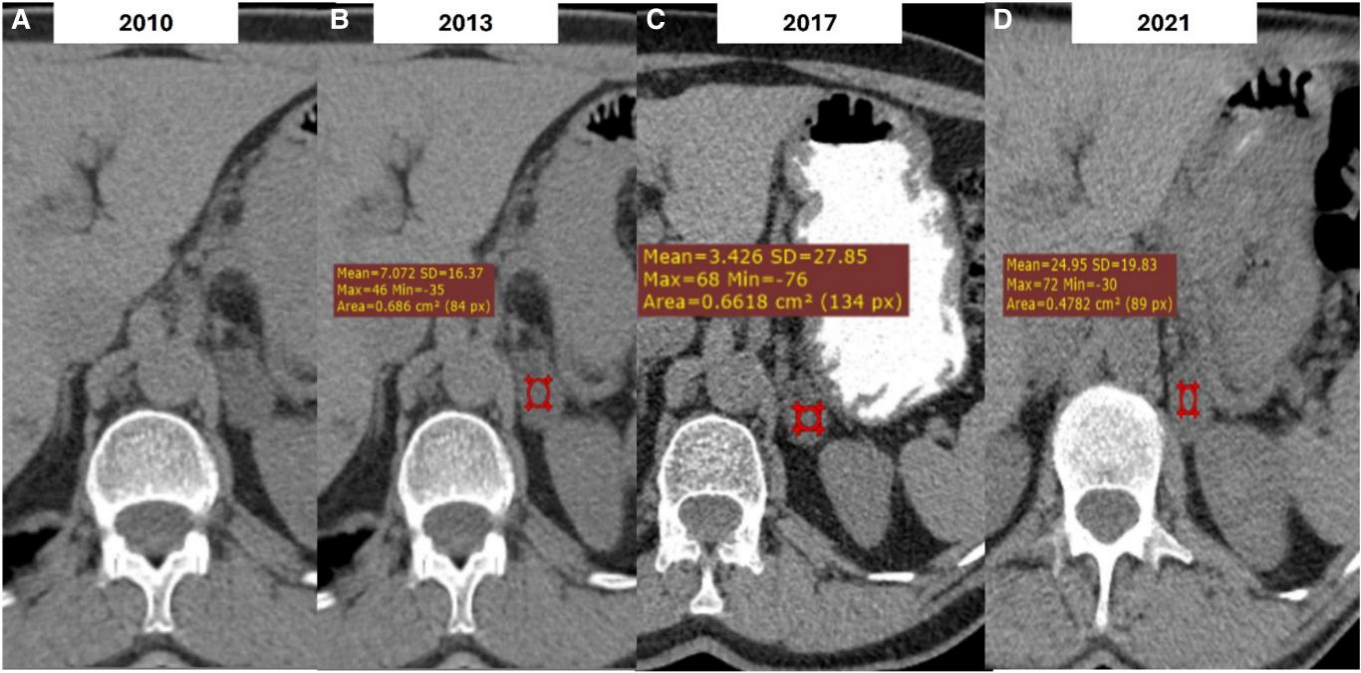
Axial unenhanced Computerised Tomography (CT) image of the upper abdomen demonstrates a nodular left adrenal lesion of 3 cm in size with low attenuation values <10 HU consistent with adenoma (A). Follow-up CT images of the same patient show no significant change of the lesion's size or density with mean ± SD density values equal to 7 ± 16.37 HU (max = 46, min = −35) (B) and 3.4 ± 27.85 HU (max = 68, min = −76) (C). On the follow-up CT performed one year before adrenocortical carcinoma (ACC) diagnosis the lesion remains stable in size but demonstrates higher mean ± SD density values equal to 24 ± 19.83 HU (D). Red circle: region of interest (ROI), HU: Hounsfield unit, Mean: mean HU, Max: maximum HU, Min: minimum HU, SD: standard deviation, Area: area of a square (cm^2^).

**Figure 2.**
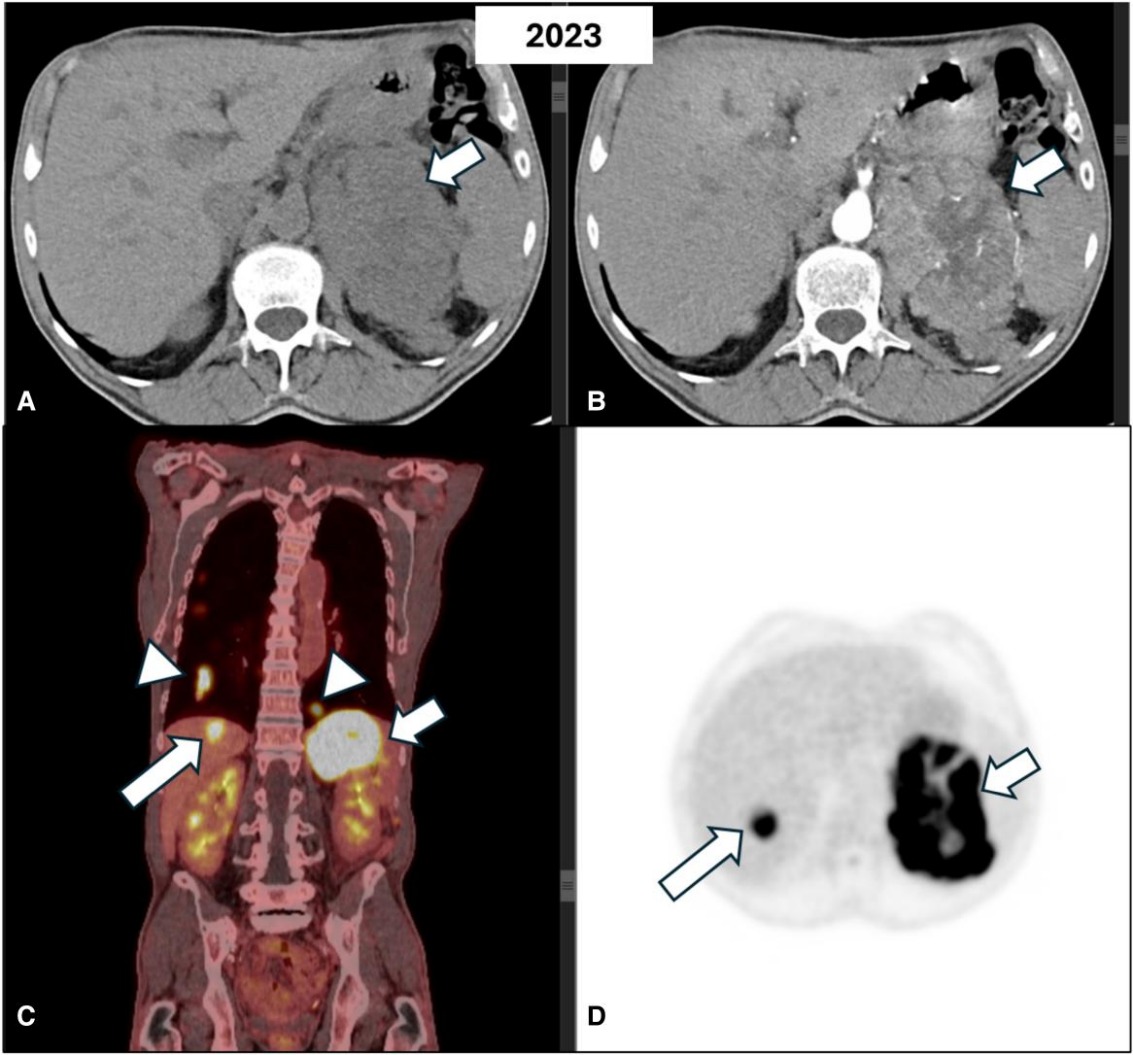
Axial computed tomography of the abdomen of the same patient before (A) and after (B) intravenous contrast administration shows a heterogeneous mass with central necrotic foci involving the left adrenal gland and extending to the surrounding tissues (arrows). On ^18^FDG-PET images (C-D), the lesion demonstrates avid ^18^FDG uptake (small arrow) and high metabolic activity lesions in the liver (long arrow) and the lungs (arrowheads) suspicious of metastases. Abbreviations: ^18^FDG-PET, 18F-fluorodeoxyglucose positron emission tomography.

**Figure 3.**
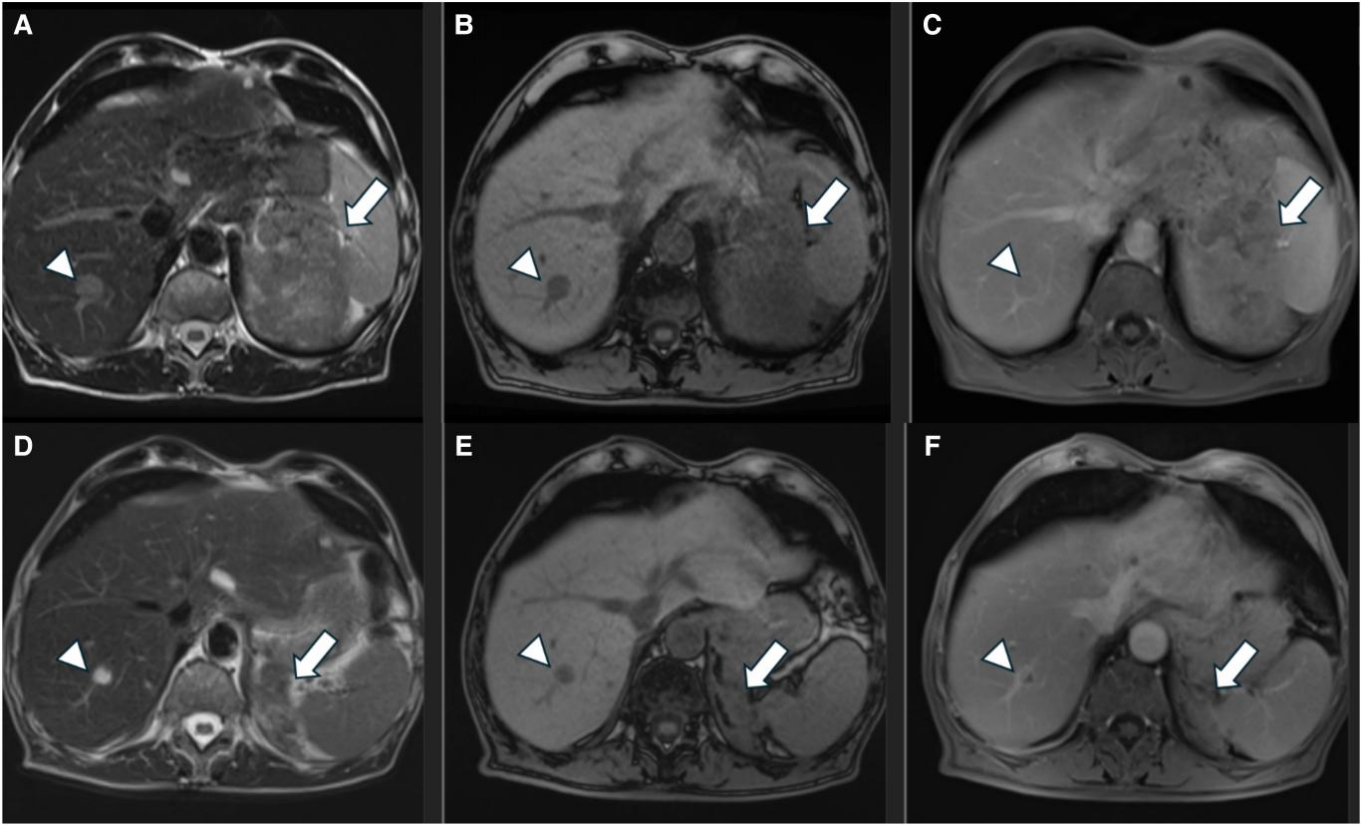
Pre-and 6 months posttreatment MRI of the same patient. Axial T2W STIR (A), T1-opposed phase (B), and contrast-enhanced T1W SPIR (C) images on pretreatment MRI demonstrate a heterogeneous left adrenal mass (arrow in A and B) with intense contrast enhancement (arrow in C). A hepatic metastasis is also seen in the right liver lobe (arrowheads in A-C). Corresponding posttreatment MRI exhibits a significant decrease in the adrenal lesion size (arrows in D-F). Note the minimal enhancement of the lesion on the postcontrast image (arrow in F), another finding indicative of the tumor's response to treatment. Liver lesion is also decreased (arrowheads in D and E) and less enhanced (arrowhead in F) on posttreatment MRI. Abbreviations: MRI, magnetic resonance imaging.

**Figure 4.**
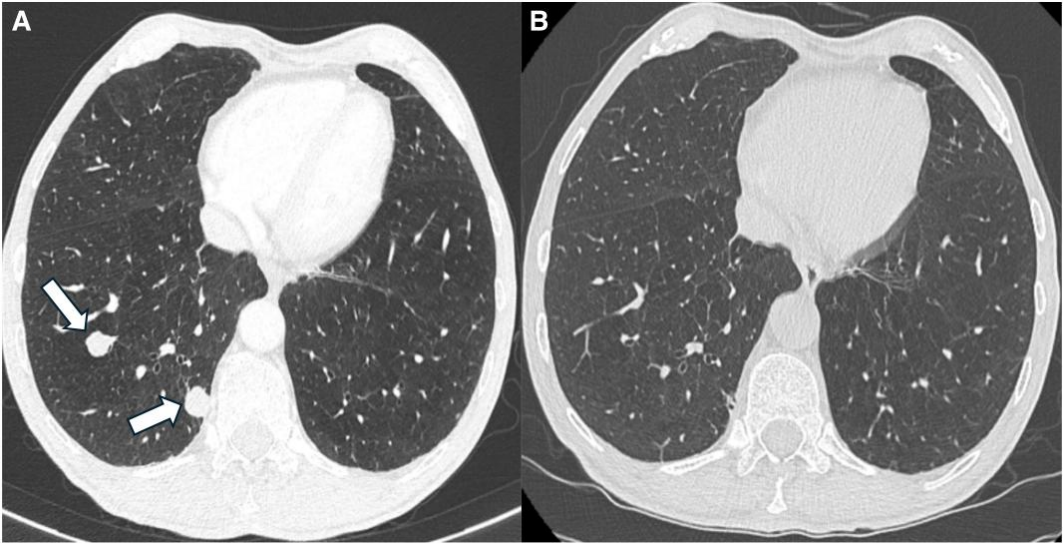
Pre-and posttreatment CT of the lung of the patient. Axial CT image at the initial presentation (A) demonstrates 2 well-circumscribed pulmonary nodules in the right lower lobe (arrows) consistent with metastases. Corresponding CT image at the same level (B), 6 months posttreatment, shows complete resolution of the metastatic nodules. Abbreviations: CT, computed tomography.

A 64-year-old male smoker with a history of hypertension was diagnosed incidentally with a 3 cm homogenous lesion of the left adrenal following an abdominal CT performed for abdominal discomfort in 2010. The lesion had a density of <10 HU and thus was in favor of an ACA. Hormonal workup showed no evidence of hormonal hypersecretion (Table 1). Since then, the patient has undergone abdominal imaging yearly for the first 3 years and then every 2 years. The size and the imaging characteristics of the lesion remained stable until 2017 when he was lost to follow-up for the next 4 years. A CT scan performed in 2021 showed a stable lesion of 3 cm albeit with higher unenhanced attenuation values of 24 HU (Fig. 1). Hormonal screening revealed no hormonal hypersecretion. However, 2 years later he presented with significant weight loss during a 3-month period, associated with fatigue and abdominal pain. An abdominal CT showed a heterogeneous left adrenal lesion measuring 12 × 11.5 × 8.6 cm exhibiting diffuse infiltration to the surrounding structures (Fig. 2). MRI confirmed the presence of an enlarged heterogeneous left adrenal mass with intense contrast enhancement (Fig. 3A-3C). In addition, 2 new suspicious lesions were described in the liver, 1 of 2 cm in segment VIII and another of 7.7 mm in segment VI, in favor of metastatic disease (Fig. 3A-3C). A thoracic CT also showed multiple secondary bilateral pulmonary lesions (Fig. 4A). 18F-fluorodeoxyglucose positron emission tomography (^18^FDG-PET) showed increased uptake of the adrenal lesion (18 SUVmax), of the hepatic and pulmonary lesions (10-12 SUVmax) and of the left adrenal vein (12 SUVmax) consistent with a vascular thrombus (Fig. 2).

**Table 1.** Hormonal functional test of the patient at the initial diagnosis of AI at the time of ACC diagnosis as well as 6 months postchemotherapy

Biochemical parameters	Reference range and units	Initial diagnosis	Follow-up 2 years before ACC diagnosis*^a^*	13 years of follow-up	6 months posttreatment
Cortisol (am)	6-17 μg/dL (165-468 nmol/L)	14.8 μg/dL (408 nmol/L)	ND	15.30 μg/dL (422 nmol/L)	12 μg/dL (331 nmol/L)
ACTH	15-57 pg/mL (3.3-12.5 pmol/L)	18.3 pg/mL (4 pmol/L)	ND	27 pg/mL (5.9 pmol/L)	248 pg/mL (54.6 pmol/L)
DHEAS	33-249 μg/dL (0.89-6.7 μmol/L)	58 μg/dL (1.5 μmol/L)	42 μg/dL (1.1 μmol/L)	410 μg/dL (11 μmol/L)	8.9 μg/dL (0.2 μmol/L)
17-OH progesterone	0.5-2.1 ng/mL (1.5-6.35 nmol/L)	1.4 ng/mL (4.2 nmol/L)	ND	33.8 ng/mL (102 nmol/L)	1.07 ng/mL (3.2 nmol/L)
Aldosterone	2-9 ng/dL (55-250 pmol/L)	9.16 ng/dL (254 pmol/L)	ND	9.7 ng/dL (269 pmol/L)	4.37 ng/dL (119 pmol/L)
PRA	0.2-2.35 ng/mL/hour	1.34 ng/mL/hour	ND	1.2 ng/mL/hour	0.2 ng/mL/hour
Testosterone	1.9-7.7 ng/mL (5.8-26.7 nmol/L)	5.9 ng/mL (20.4 nmol/L)	ND	2.2 ng/mL (7.6 nmol/L)	0.7 ng/mL (2.63 nmol/L)
SHBG	190-722 μg/dL (20-76 nmol/L)	ND	ND	313.5 μg/dL (33 nmol/L)	1900 μg/dL/L (>200 nmol)
Δ4 androstenedione	0.6-3.3 ng/mL (2.09-11.5 nmol/L)	ND	ND	4.3 ng/mL (15 nmol/L)	0.82 ng/mL (2.8 nmol/L)
UFC	20-133 μg/24h (55-365 nmol/24 hours)	100.5 μg/24 hours (276 nmol/24 hours)	67.6 μg/24h (182.5 nmol/24 hours)	203 μg/24h (560 nmol/24 hours)	179 μg/24 hours (494 nmol/24 hours)
Cortisol (1 mg ODST)	<1.8 μg/dL (49 nmol/L)	1.7 μg/dL (46.8 nmol/L)	1.8 μg/dL (49 nmol/L)	9 μg/dL (248 nmol/L)	ND

Abbreviations: AI, adrenal incidentaloma; ACC, adrenocortical adenoma; DHEAS, sulfate dehydroepiandrosterone; ND, no data; ODST, overnight dexamethasone suppression test.; PRA, plasma renin activity; UFC, urinary free cortisol.

^*a*^At the time of imaging changes (increased Hounsfield units).

## Diagnostic Assessment

**Figure 5.**
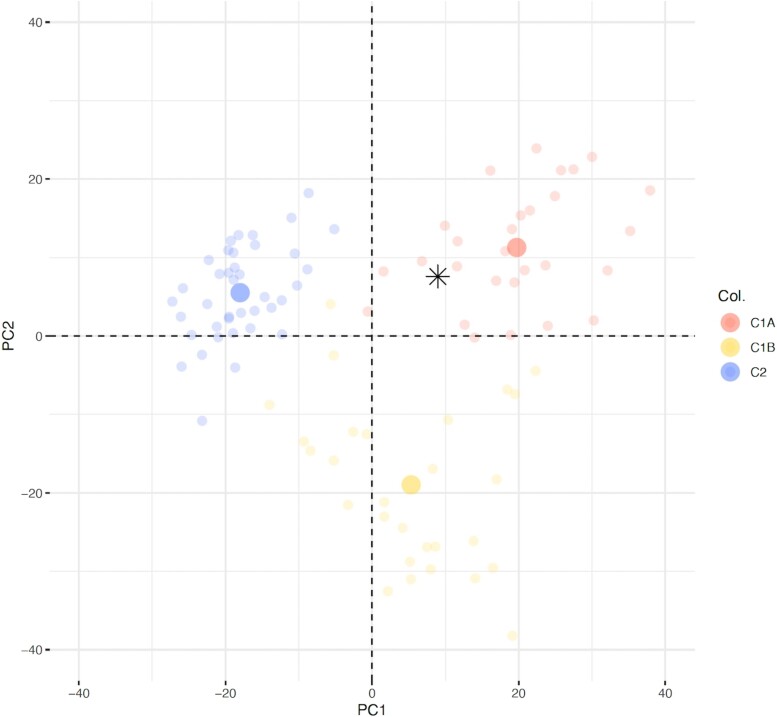
Transcriptome classification. Patient's sample (*) was projected on the two principle components (Dim1, Dim2) of a principal component analysis (PCA) in a reference cohort of 95 patients (14). Samples from this reference cohort are presented as faint circles colored by transcriptome class: blue for adrenocortical adenomas “C2”, red for adrenocortical carcinoma of poor prognosis “C1A” and yellow for adrenocortical carcinoma of better prognosis “C1B”.

Clinically the patient was cachectic and normotensive without any signs of Cushing syndrome. The hormonal profile demonstrated excessive cortisol and androgen secretion (Table 1). A biopsy of 1 of the liver lesions confirmed metastasis from a high-grade ACC with a Ki-67 labeling index (LI) of 70%. Immunohistochemical analyses were positive for steroidogenic factor 1, synaptophysin+, and melan A+ and negative for pankeratin, epithelial membrane antigen, and chromogranin A. Molecular classification based on transcriptome analysis of the hepatic metastasis was performed using 3′ RNA-sequencing from formalin-fixed paraffin-embedded sample, as previously described (14). The sample was classified as “C1A,” which is considered predictive of a poor outcome **(**Fig. 5).

## Treatment

The case was presented to the multidisciplinary board where it was decided that no surgical operation was feasible and the patient was started on high-dose mitotane (maximum 6 g) treatment, achieving levels of 22.5 mg/L within 8 weeks albeit with significant side effects necessitating titration of the mitotane dose to 4.5 g daily. Replacement treatment with hydrocortisone along with fludrocortisone was also needed. The patient received concomitantly 6 cycles of chemotherapy (etoposide, doxorubicin, and cisplatin) based on the FIRMACT study (15).

## Outcome and Follow-up

Grade 3 side effects including nausea, fatigue, instability, neurosensorial symptoms, and difficulties in memory and concentration developed 2 to 3 weeks after the initiation of both mitotane and chemotherapy treatments. In addition, the patient developed an osteoporotic right colles fracture necessitating the administration of denosumab.

Imaging 3 months postinitiation of both treatments showed a partial response of all lesions and at 6 months a further partial response based on RECIST 1.1 criteria of the adrenal and the hepatic lesions (Fig. 3D-3F) with complete resolution of the lung metastases (Fig. 4B). The patient is still alive 8 months after ACC diagnosis and continues only on mitotane treatment due to his poor performance status.

## Discussion

We describe a rare case of a patient diagnosed initially with a 3 cm nonfunctional ACA that transformed after 13 years of follow-up to a metastatic ACC. Although it is not possible to define the exact timing of transformation, it most likely occurred 11 years after the initial diagnosis of ACA and 2 years before the diagnosis of the ACC when a change in the enhanced attenuation value occurred besides the lesion being stable in size.

Recent European Society of Endocrinology clinical practice guidelines for the management of AIs suggest against any further imaging of adrenal masses <4 cm in size with clearly benign radiological features that are functionally inactive (4). However, in patients with indeterminate radiological findings, further imaging after 6 to 12 months is suggested, with either CT/MRI or even ^18^FDG-PET. Thus, in the present case, the initial diagnosis of a benign nonfunctioning ACA should have been made and no further follow-up would have been suggested.

A literature search through PubMed identified 9 additional ACC cases reported from 2017 to 2024 that were initially diagnosed as ACA of <4 cm with mostly benign imaging characteristics that transformed to ACCs (Table 2) (5-13). Hormonal investigations demonstrated that 3 were functional (6, 8, 11). All cases were initially evaluated with a CT scan, and in 3 cases an MRI was additionally performed (6, 11, 13). Six cases had typical characteristics of ACAs on CT scan exhibiting low unenhanced attenuation values of <10 HU (5, 7, 8, 9, 10, 11) whereas in the remaining 3 (6, 12, 13), the findings were ambiguous (Table 2).

**Table 2.** Results of the literature review including 10 case reports of initially benign AIs transformed to ACCs

Ref	Age, Sex(F/M)	Initial symptom, lateralisation	Initial size	Initial CT/MRI features	Initial hormonal profile	Last CT/MRI before ACC diagnosis: time/size	ACC characteristics on imaging (CT/MRI)	Time to progression from initial diagnosis	Final size, increase per year	Hormonal hypersecretion on FU	Histology
Parry et al 2024 (5)	70 y F	Flushing, right AI	8 mm	3 HU (noncontrast)/ND	NF	2y/8 mm ACA (stable for 6 y)	32 HU noncontrast (washout = 0%)	7 y	66 × 49 mm, 29 mm/y	NF	ACC, Weiss:5, Ki-67:20%, (stage II)
Ohkubo et al 2024 (6)	50 y, F	Fever, right AI	20 × 20 mm	32 HU (noncontrast), delayed washout/lipid-rich	Hyperaldosteronism	ND	Enlarged size	1 y	130 mm, 110 mm/y	Cortisol and aldosterone	ACC, Weiss:7, Ki-67:36%
Kohli et al 2021 (7)	70 y, F	No relevant symptoms, left AI	20 × 16 mm	>10 HU (noncontrast) with 67% absolute washout and 47% relative wash out/ND	NF	1y/20 × 16 mm ACA (stable for 7 y)	37 HU noncontrast, 98 HU postcontrast	8 y	58 × 43 mm, 38 mm/y	Cortisol	ACC, Ki-67:30%, (stage II)
Aono et al 2022 (12)	77 y, F	No relevant symptoms, left AI	15 × 16 × 15 mm	30 HU, homogeneous, rounded/ND	NF	1y/21 × 28 × 30 mm ACA (stable for 6 y)	Heterogenous with cystic degeneration/No signal loss in the out of phase	9 y	35 × 41 × 54 m, 14 mm/y	Cortisol	ACC, Weiss:4, Ki-67:20%, *CTNNB1, G34A* mutation
Gagnon et al 2020 (13)	32 y, F	Non specific abdominal pain, left AI	29 × 19 mm	31 HU/isointense in T2 with few hyperintense areas, no loss of signal in the out of phase	NF	6y/29 × 19 mm ACA (stable for 5 y)	Two new hepatic lesions	10 y	90 × 82 mm, 10 mm/y	Cortisol and androgens	ACC, Ki-67:30%, *APC* mutation
Rebielak et al 2019 (8)	28 y, F	Left flank pain, left AI	27 × 21 mm	In favor of ACA/ND	Elevated total/free testosterone	7y/27 × 21 mm ACA (stable for 7 y)	25% absolute washout/47 × 59 mm enhanced left adrenal mass	7 y	56 × 37 × 40 mm, 4 mm/y	Testosterone	ACC, Ki-67:30%, (stage II)
Barsukova et al 2019 (11)	72 y, F	ND, left AI	−2007: 15 mm -2016: 37 mm -2018: 68mm	−2007: In favor of ACA/ND -2016: 15 HU with 42% relative washout/ND -2018: ND/large lobulated heterogeneous mass	2007:NF 2016: hypercortisolaemia 2018: recurrence of hypercortisolaemia (adrenalectomy)	2 y (post-adrenalectomy)	Heterogeneous with new nodules superior and posterior to kidney	−NF to cortisol-secreting ACA: 9 y - Recurrence postsurgery: 2 y	68 × 41 × 59 mm, 34 mm/y	Cortisol	−1st surgery Weiss:2 (ACA) -2nd surgery: ACC
Thuzar et al 2018 (9)	47 y, F	No relevant symptoms, left AI	13mm	In favor of ACA/ND	NF	10 m/20 × 15 × 17mm	Large heterogenous atypical mass	3 y	100 × 90 × 130 mm, 80 mm/y	NF	ACC, Weiss:5, Ki-67:40%, (stage II)
Belmihoub et al 2017 (10)	71 y, M	Urinary tract infection, right AI	17 mm	In favor of ACA/ND	NF	5y/21 mm (9 HU)	5 HU noncontrast, < 50% absolute washout/No signal loss in the out-of-phase	8 y	60 mm, 8 mm/y	Cortisol	ACC, Weiss:8, Ki-67:30% (stage II)

Abbreviations: ACA, adrenocortical adenoma; ACC, adrenocortical carcinoma; AI, adrenal incidentaloma; CT, computerized tomography; F, female; FU, follow-up; HU, Hounsfield units; M, male; MRI, magnetic resonance imaging; ND, no data; NF, nonfunctioning; y, years.

A further retrospective study of 439 ACCs demonstrated that 20 were characterized as AIs with a median initial size of 2.8 cm (16). However, all presented atypical CT characteristics, with ether median precontrast density of 36 HU or heterogeneous adrenal mass or calcification (17). Another retrospective study, including 422 ACCs, showed that 20 had previously been diagnosed as ACAs measuring <4 cm in size. However, only 2/20 ACCs exhibited imaging characteristics suggestive of a benign lesion (17).

Recently, it was shown that a growth rate of 3 mm/year could distinguish ACA from malignant lesions with 100% sensitivity and specificity (18). However, 7 cases in the literature presenting initially with ACAs that remained stable in size for the first 5 to 7 years were diagnosed as ACCs after 7 to 10 years from the initial diagnosis of ACAs (5, 7, 8, 10, 11, 12, 13). Thus, surveillance with imaging during only the first 5 years from initial diagnosis would have missed the malignant transformation of these ACAs to ACCs. Moreover, a retrospective analysis showed that the combination of an adrenal tumor size of 3 cm with an unenhanced attenuation value of 20 HU or of 4 cm with an unenhanced attenuation value of 15 HU was associated with a 100% positive predictive value for the diagnosis of ACA (19).

The transformation of an initially denoted ACA to ACC has been described not only with respect to imaging characteristics but also their hormonal profile. Five tumors initially characterized as nonfunctioning ACAs transformed to cortisol-secreting ACCs similar to our case (6, 7, 10, 12, 13).

In 8 of the 9 described cases, surgery was the first line treatment and histology demonstrated in 7 of them high-grade ACCs. Our ACC was also classified as high-grade ACC with a Ki-67 LI value of 70%.

The transformation of ACA to ACC is largely questioned due to the increased frequency of the former and the rarity of the latter entity. Whether ACCs develop from benign adenomas or represent a de novo event has not been fully clarified. Some authors support the hypothesis of the independent development of 2 distinct tumors, derived from separate clones in the same adrenal gland that are histologically distinguishable, with 1 central malignant tissue and an adjacent benign adrenal tissue (20, 21). However, the absence of benign tissue could be explained by the aggressivity and the invasion of the malignant tumor as described in 1 case report (10). The second hypothesis suggests that adrenal tumorigenesis is a progressive multistep procedure transforming normal adrenal tissue to ACA and eventually to ACC (22).

Recently, several prognostic scores have been developed based on histopathological markers, such as Ki-67 LI, number of mitoses (Helsinki score), or combining histological and clinical variants (S-GRAS score) (23). The genetic analysis seems to present the cornerstone of the progress made regarding the prediction of ACC behavior. Several molecular parameters such as Wnt/β-catenin pathway alterations increased p53 expression, insulin growth factor-II overexpression, hypermethylation of CpG loci, and overexpression of several miRNAs allow classifying these tumors based on their genetic profile either in the C1A (poor outcome ACC) or C1B (better outcome ACC) group (14, 24) (Fig. 5).

A large series including 512 ACC cases reported that 38% were diagnosed incidentally with a frequency increasing with age (25). In contrast to a previous retrospective study (17), ACCs found incidentally were less aggressive, presenting also better prognosis compared with the symptomatic ones (25). The clinical history of the 10 cases initially diagnosed as benign ACAs showed that only half were completely asymptomatic and could be characterized as “true AI.” In 3 patients (7, 9, 12) as in our case, a pre-existing atypical abdominal symptom led to the performance of further imaging, whereas in 1 case, this information was not available (11).

A meta-analysis performed almost 15 years ago showed that the risk of an AI developing into a functional ACA or an ACC during follow-up is rare accounting for <1% and 0.2% of cases, respectively (26). Most importantly, during the follow-up, the risk of false-positive cases was 50 times greater than the true-positive ones. Additionally, the average radiation exposure during a CT scan was associated with a 1/430 to 2170 chance of developing cancer similar to the likelihood of developing adrenal malignancy during a 3-year follow-up of AIs (26).

In conclusion, ACC is a rare disease, and ACC originating from an ACA is an even rarer event, but it does exist. Imaging follow-up of AIs with benign imaging characteristics has been modified according to recent guidelines to avoid excessive investigations. Considering the potential implications of continuous monitoring and unnecessary radiation exposure to identify patients who could be at risk for developing ACCs, additional predictive biomarkers need to be developed to potentially identify patients who may exhibit such a course.

## Learning Points

- ACC incidence among AI is generally rare, and transformation of an ACA to an ACC is extremely rare but exists.
- No robust radiological features are currently available that can help to predict ACA at risk of transforming to ACC.
- The identification of such predictive biomarkers presents an unmet need and should probably be based on the genetic profile of these tumors.

## Data Availability

Some or all datasets generated during and/or analyzed during the current study are not publicly available but are available from the corresponding author on reasonable request.
